# Catching a Liar Through Facial Expression of Fear

**DOI:** 10.3389/fpsyg.2021.675097

**Published:** 2021-06-08

**Authors:** Xunbing Shen, Gaojie Fan, Caoyuan Niu, Zhencai Chen

**Affiliations:** ^1^Department of Psychology, Jiangxi University of Chinese Medicine, Nanchang, China; ^2^Beck Visual Cognition Laboratory, Louisiana State University, Baton Rouge, LA, United States

**Keywords:** deception, leakage theory, fear, machine learning, asymmetry

## Abstract

High stakes can be stressful whether one is telling the truth or lying. However, liars can feel extra fear from worrying to be discovered than truth-tellers, and according to the “leakage theory,” the fear is almost impossible to be repressed. Therefore, we assumed that analyzing the facial expression of fear could reveal deceits. Detecting and analyzing the subtle leaked fear facial expressions is a challenging task for laypeople. It is, however, a relatively easy job for computer vision and machine learning. To test the hypothesis, we analyzed video clips from a game show “The moment of truth” by using OpenFace (for outputting the Action Units (AUs) of fear and face landmarks) and WEKA (for classifying the video clips in which the players were lying or telling the truth). The results showed that some algorithms achieved an accuracy of >80% merely using AUs of fear. Besides, the total duration of AU20 of fear was found to be shorter under the lying condition than that from the truth-telling condition. Further analysis found that the reason for a shorter duration in the lying condition was that the time window from peak to offset of AU20 under the lying condition was less than that under the truth-telling condition. The results also showed that facial movements around the eyes were more asymmetrical when people are telling lies. All the results suggested that facial clues can be used to detect deception, and fear could be a cue for distinguishing liars from truth-tellers.

## Introduction

Are there any observable behaviors or cues that can differentiate lying from truth-telling? Almost all researchers in the field of deception detection agree that there is no “Pinocchio's nose” that can serve as an easy indicator of deception (DePaulo et al., 2003). Nevertheless, many researchers are still trying to find cues to deception (Levine, 2018; Denault et al., 2020). The “leakage theory” asserts that high-stake lies (the rewards come with serious consequences or there can be severe punishments) can result in “leakage” of the deception into physiological changes or behaviors (especially microexpressions that last for 1/25 to 1/5 s; Ekman and Friesen, 1969; Ekman, 2003; Porter et al., 2011, 2012; Su and Levine, 2016; Matsumoto and Hwang, 2020). Specifically, from the perspective of leakage theory (ten Brinke and Porter, 2012; Ten Brinke et al., 2012a,b), observable emotional facial expressions (microexpressions and macroexpressions) can, to some degree, determine who is lying and who is telling the truth (It is a probability problem (see Levine, 2018, 2019). However, debate exists for this possibility. While some researchers (ten Brinke and Porter, 2012; Ten Brinke et al., 2012b; Matsumoto and Hwang, 2018) argued that emotional facial microexpression could be a cue to lies supported their claims by empirical evidence, Burgoon (2018) argued that detecting microexpressions is not the best way of catching liars. Furthermore, Vrij et al. (2019) even categorized microexpression into pseudoscience.

Even if it can be difficult, or even impossible for human beings to detect liars based on microexpressions, there do exist some behavioral cues that can, to some degree, differentiate lying from truth-telling (Vrij et al., 2000, 2006). Specially, pupil dilation and pitch are shown to be closely related to lying (Levine, 2018, 2019). Most of the deception researchers agree that lying involves processes or factors such as arousal and felt emotion (Zuckerman et al., 1981). Therefore, emotional facial expressions can be valid behavioral cues to deception. Meanwhile, there are involuntary aspects of emotional expression. As noted by Darwin, some actions of facial muscles were the most difficult to be voluntarily controlled and were the hardest to be inhibited (the so-called Inhibition Hypothesis (see also Ekman, 2003). When a strongly felt genuine emotion is present, the related facial expressions cannot be suppressed (Baker et al., 2016). Hurley and Frank (2011) provided evidence for Darwin's hypothesis and found that deceivers could not control some particular elements of their facial expression, such as eyebrow movements. The liars would feel fear, duping delight, disgust, or appear tense while lying, and would attempt to suppress these emotions by neutralizing, masking, or simulating (Porter and Ten Brinke, 2008). However, the liars could not inhibit them completely and the felt emotion would be “leaked” out in the form of microexpressions, especially under high-stake situations (Ekman and Friesen, 1969).

The claim of emotional leakage is supported by some recent research (Porter et al., 2011, 2012). When liars fake an unfelt emotional facial expression, or neutralize a felt emotion, at least one inconsistent expression would leak and appear transiently (Porter and Ten Brinke, 2008). ten Brinke and Porter (2012) showed that liars would present unsuccessful emotional masking and certain leaked facial expressions (e.g., “the presence of a smirk”). In addition, they found that false remorse was associated with (involuntary and inconsistent) facial expressions of happiness and disgust (Ten Brinke et al., 2012a).

In addition to the support for emotional leakage, research also shows that leaked emotions can differentiate lies and truth-telling. Wright Whelan et al. (2014) considered a few cues that had successfully told liars and truth-tellers, including gaze aversion and head shakes. They combined the information from each cue to classify individual cases and achieved an accuracy rate as high as 78%. Meanwhile, Wright Whelan et al. (2015) found non-police and police observers could reach an accuracy of 68 and 72%, respectively, when required to detect deception in high-stake, real-life situation. Matsumoto and Hwang (2018) found that facial expressions of negative emotions that occurred for <0.40 and 0.50 s could differentiate truth-tellers and liars. These studies all suggested that leaked facial expressions could help human beings detect liars successfully.

Besides human research, attempts have also been made to use machine learning to automatically detect deception by utilizing leaked emotions. A meta-analysis by Bond and DePaulo (2006) showed that human observers only achieved a slightly-better-than-chance accuracy when detecting liars. Compared to humans, some previous works with machine learning used the so-called reliable facial expressions (or involuntary facial expressions) to automatically detect deceit and achieved an accuracy above 70% (Slowe and Govindaraju, 2007; Zhang et al., 2007). Given that the subtle differences of emotional facial expressions may not be detected by naïve human observers, computer vision may capture the different and subtle features between lying and truth-telling situations that cannot be perceived by a human being. Su and Levine (2016) found that emotional facial expressions (including microexpressions) could be effective cues for machine learning to detect high-stake lies, in which the accuracy was much higher than those reported in previous studies (e.g., Bond and DePaulo, 2006). They found some Action Units (AU, the contraction or relaxation of one or more muscles (see Ekman and Friesen, 1976), such as AU1, AU2, AU4, AU12, AU15, and AU45 (blink), could be potential indicators for distinguishing liars from truth-tellers in high-stake situations. Bartlett et al. (2014) showed that computer vision could differentiate deceptive pain facial signals from genuine pain facial signals at 85% accuracy. Barathi (2016) developed a system that detected a liar based on facial microexpressions, body language, and speech analysis. They found that the efficiency of the facial microexpression detector was 82%. Similarly, the automated deception detection system developed by Wu et al. (2018) showed that predictions of microexpressions could be used as features for deception detection, and the system obtained an area under the precision-recall curve (AUC) of 0.877 while using various classifiers.

The leakage theory of deception predicts that when lying, especially in high-stake situations, people would be afraid of their lies being detected and therefore result in fear emotions. These fear emotions could then leak and have the potential to be detected (Levine, 2019). Meanwhile, it is presumed that if the fear associated with deception is leaked, the duration of the leaked fear would be shorter due to the nature of leaking and repressing (which would be presented as fleeting fear microexpressions). Some may argue that the fear emotions may also appear in truth-telling. It can be true. Nevertheless, for a truth-teller, the fear of being wrongly treated as a liar would be less leaking, since a truth-teller does not need to try hard to repress the fear as liars do. As a result, the degree of repressing will be different between liars and truth-tellers. On average, the duration of fear (or AUs of fear) in lying situations would be shorter than that in truth-telling situations due to the harder repressing in the former ones.

Stakes may play a vital role while using an emotional facial expression as a cue to detect deception. Participants experience fewer emotions or less cognitive load in laboratory studies (Buckley, 2012). Almost all laboratory experiments are typical of low stakes and are not sufficiently motivating to trigger emotions giving rise to leakage (in the form of microexpressions). Consequently, liars in laboratory experiments are not as nervous as in real-life high-stake situations, with no or little emotion leakage. As noted by Vrij (2004), some laboratory-based studies in which the stakes were manipulated showed that high-stake lies were easier to detect than low-stake ones. Frank and Ekman (1997) stated that “*the presence of high stakes is central to liars feeling strong emotion when lying*.” Therefore, lying in high-stake situations would be more detectable by using emotional facial expression cues, and leaked emotional facial expressions would mostly occur in a high-stake context.

Hartwig and Bond (2014) had an opposite opinion and argued that even in high-stake situations, it could still be difficult to tell liars from truth-tellers. They claimed that the context of the high stake would influence both liars and truth-tellers, as liars and truth-tellers might experience similar psychological processes. In other words, high-stake situations would cause inconsistent emotional expressions, like fear, not only in liars, but also in truth-tellers. This claim is true to some degree (ten Brinke and Porter, 2012), but high stakes do not necessarily eliminate all the differences between liars and truth-tellers. Even though high-stake situations increase pressure on both liars and truth-tellers, it can be assumed that the degree of increment would be different, and liars would feel much higher pressure than truth-tellers under high stakes. In addition, fabricating a lie requires liars to think more and therefore would cause a higher emotional arousal in them than in truth-tellers. Consequently, for liars, the frequency or probability of leaking an inconsistent emotional expression (say, fear) would be higher and thus easier to detect. In theory, the higher the stakes are, the more likely cues associated with deception (e.g., fear) are leaked, and the easier the liars could be identified using these cues.

Besides duration, other dynamic features (Ekman et al., 1981; Frank et al., 1993) could also vary in genuine and fake facial expressions, such as symmetry. Ekman et al. (1981) manually analyzed the facial asymmetry by using the Facial Action Coding System (FACS) and showed that genuine smiles have more symmetry when compared to a deliberate smile. Similarly, the leaked emotional facial expressions of fear while lying and the less leaked ones when telling a truth may also show different degrees of symmetry. However, the approach Ekman et al. (1981) used could be time-consuming and subjective. Thus, in the current study, we proposed a method that used coherence (a measure of the correlation between two signals/variables) to measure the asymmetry. The more symmetrical the facial movements of the left and right face, the higher the coefficient of correlation between them. Consequently, the value of coherence (ranges from 0 to 1) can be a measurement of asymmetry or symmetry.

Based on the leakage theory and previous evidence, we hypothesize that (1) emotional facial expressions of fear (fear of being caught) can differentiate lying from truth-telling in high-stake situations; (2) the duration of AUs of fear in lying would be shorter than that in truth-telling; (3) the symmetry of facial movements will be different, as facial movements in lying situations will be more asymmetrical (due to the nature of repressing and leaking).

## Methods

### The Database

The database we used were 32 video clips of 16 individuals telling lies in half of them and truth in the other half. All of the video clips were recorded in a high-stake game show. The reason we used the current design was that cues to detect deception could differ from person to person, and what spotted one liar was usually different from the signals that revealed the next liar (Levine, 2019). Consequently, cues may vary from sender to sender. The same person, however, would display almost the same facial expression pattern on different occasions. Therefore, the relatively ideal experimental materials should be composed by the same individual who tells both lies and truth to exclude or reduce the variation resulted from individual differences.

The video clips recorded individuals' facial expressions in the game show “the Moment of Truth.” Prior to the show, the contestants took a polygraph exam when they answered 50 questions. During the show, 21 of the same questions were asked again and the contestants were required to answer them in front of the studio audience. The questions became progressively more personal as the contestants moved forward (an example of an extremely personal question is: Have you ever paid for sex?). If the contestant gave the same answer to a question as they did in the polygraph exam (which means they were telling the truth), they moved on to the next question; lying (as determined by the polygraph) or refusing to answer a question ends the game (see https://en.wikipedia.org/wiki/The_Moment_of_Truth_(American_game_show) for details). During the game show, most of the people talked emotionally and showed natural emotional facial expressions because of the high-stake situations they were in. The ground truth was obtained by a pre-show polygraph test that determined whether an individual was lying or not in the game show. Meanwhile, the stakes in the game show can be high (the highest gain from the show can reach at 500,000 US dollars, and cues to deception will be more pronounced than when there was no such monetary incentive (see DePaulo et al., 2003).

Participants were eight males and eight females who ended the game with lying. That way, there was at least one lying video clip for each participant. The video clips consist of the moments when the individuals were answering the questions, that is, from the end of the questioning to the end of the answering. To simplify calculation, we merged all the truth-telling video clips for each participant into a single one, and we ended up having one video for each type, truth-telling and lying, for each person. The duration of the video clips ranges from 3 s to 280 s, with an average duration of 56.6 s. Because of the game show setting that lying ends the game, the truth-telling video clips were much longer than the lying ones (*mean* = 105.5 s for truth-telling videos, and *mean* = 7.8 s for lying videos). In total, there were 50,097 frames for truth-telling video clips and 3,689 frames for lying video clips. The median of frames is 199 for lying video clips and is 2,872.5 for truth-telling video clips, with a frame rate of 30 f/s.

### Using Computer Vision to Compare the Features in Video Clips While People Lying or Telling the Truth

Asking people to find out the cues to deception is difficult. Furthermore, naïve human observers may not be able to perceive the subtle differences of the emotional facial expressions between telling lies and telling the truth. Alternatively, computer vision may be more capable of doing so. We proposed a method aimed to use the AUs of fear to discern deceptive and honest individuals in high-stake situations.

#### Emotional Facial Expressions of Fear

We, first, imported the video clips into OpenFace (Baltrusaitis et al., 2018) to conduct computer video analysis. This software automatically detects the face, localizes the facial landmark, outputs the coordination of the landmarks, and recognizes the facial AUs. OpenFace is able to identify 18 AUs. According to Frank and Ekman (1997), telling a consequential lie results in emotions such as fear and guilt. Therefore, we focused on the AUs of fear, i.e., AU1, AU2, AU4, AU5, AU20, AU26. For each frame of videos, we obtained presence (0 or 1) and intensity (any number from 0 to 5) for each AU from OpenFace. Once we obtained the AU information from OpenFace, we then used MATLAB to calculate AUs of the emotional facial expression of fear. It was done by multiplying the output values of presence (0, 1) and the value of the intensity (from 0 to 5) for each frame. We then analyzed the AUs with statistical analysis, and also made classification predictions with machine learning.

For statistical analysis, we took the average of each AU across all frames in one condition per participant. We ended up with one AU value for each condition for each person. We then bootstrapped the data for statistical analysis.

For machine learning, we resampled the data with SMOTE before building the model. SMOTE is an over-sampling technique that solves class imbalance problem by using interpolation to increase the number of instances in the minority class (Chawla et al., 2002). Resampling was necessary because the data are unbalanced, with the video clips of truth much longer than those of deception, 50,097 frames vs. 3,689 frames. It was consistent with the real life that lying was not that frequent compared to truth-telling, but it could still affect the reliability and validity of the model.

We then used WEKA (Hall et al., 2009), a machine learning software, to classify the videos into a truth group and a deception group. Three different classifiers were trained *via* a 10-fold cross-validation procedure. The three classifiers were Random Forest, K-nearest neighbors, and Bagging. Random Forest operates by constructing a multitude of decision trees (which is also a better choice for unbalanced datasets (see Bruer et al., 2020). K-nearest neighbors (lazy.IBK in WEKA) achieves classification by identifying the nearest neighbors to a query example and using those neighbors to determine the class of the query (Cunningham and Delany, 2004). Bagging is a method for generating multiple versions of a predictor and using these to get an aggregated predictor (Breiman, 1996).

#### The Duration of Fear

We used MATLAB to count the duration of AUs of fear (the number of frames when the corresponding AU was present). Because the frame rates of all the videos were the same, the number of frames could represent the duration of AU. Then the precise duration was obtained by dividing the total number of frames by frame rate, i.e., 30.

#### The Symmetry of Facial Movements

**Figure 1:**
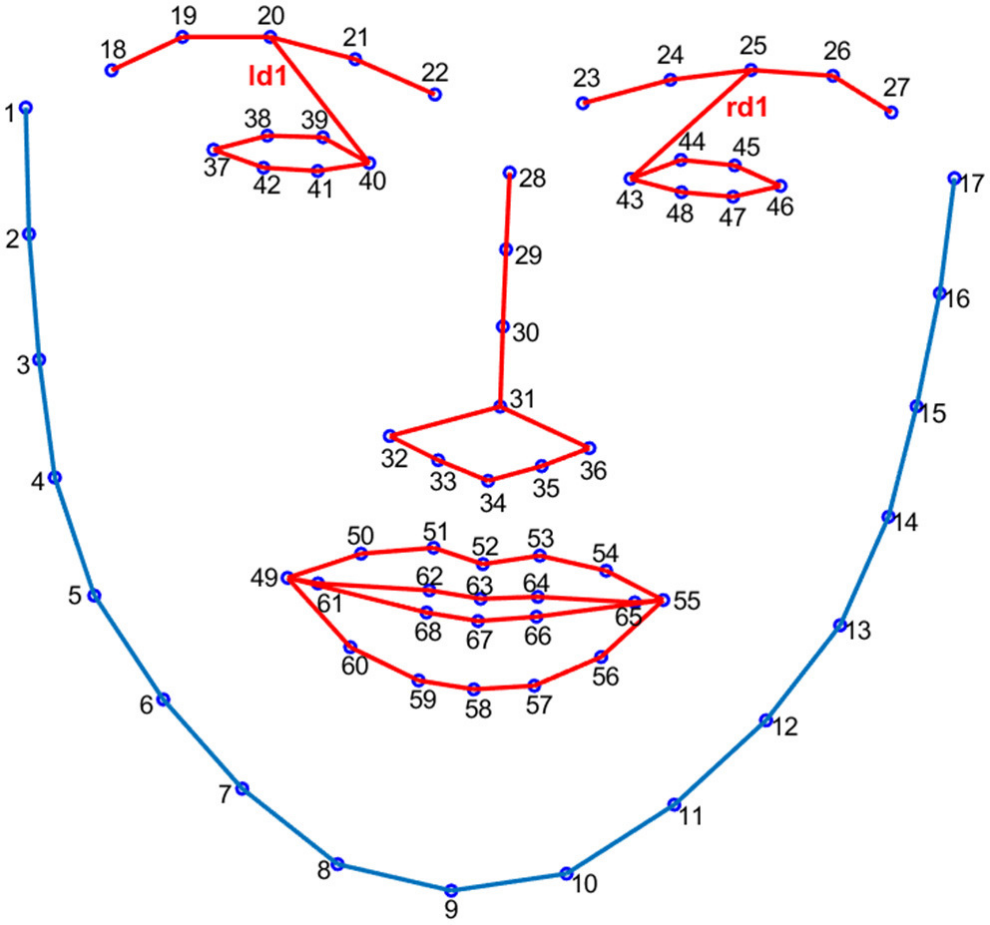
The 68 facial landmarks and the Euclidean distances of ld1 and rd1.

Beh and Goh (2019) proposed a method to detect the changes in the Euclidean distances of facial landmarks to find out microexpressions. We used the distances of ld1 and rd1, which are distances between facial landmarks at the left/right eyebrow and left/right eye (index 20/25 and index 40/43, see Figure 1), to investigate the synchronization and symmetry between left and right facial movements. The MATLAB function of Wcohenrence (wavelet coherence, the values ranged from 0 to 1) was used for this purpose, as this function returns the magnitude-squared wavelet coherence, which is a measure of the correlation between two signals (herein ld1 and rd1) in the time-frequency domain. If the left and right facial movements have perfect synchronization and symmetry, the value of wavelet coherence would be 1.

#### Summary of Data Processing

**Figure 2:**
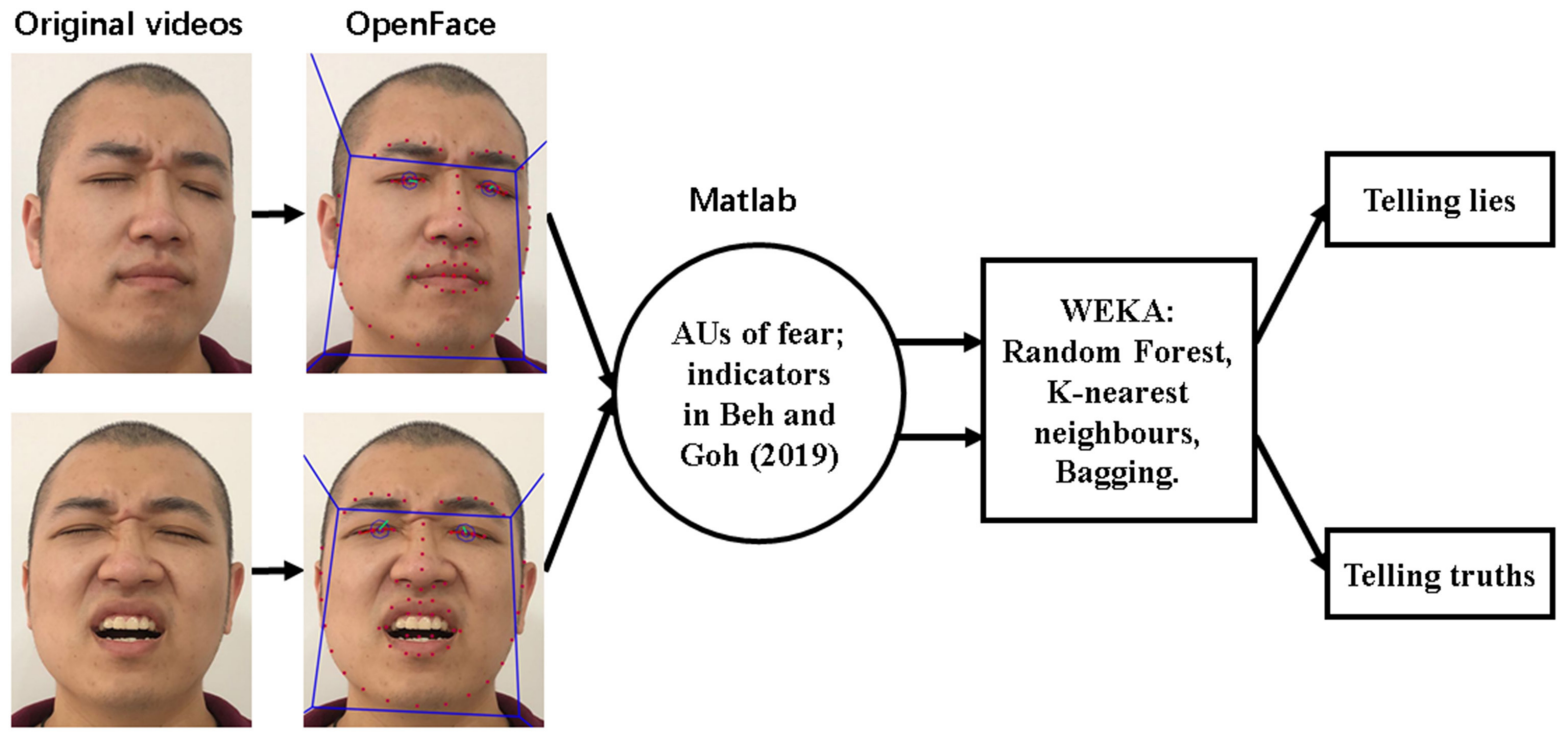
Overview of the procedure of classifying video clips. The model used here for demonstrating the processing flowchart is the third author.

All of the aforementioned steps of classifying the truth or deception in the video clips are demonstrated in Figure 2. First, OpenFace detected the face, localized the landmarks, output the presence and intensity of AUs. Following that, AUs of fear, as well as indicators used to calculate symmetry in each frame from both lying and truth video clips, were merged into a facial movement description vector (frame by frame). Finally, in the classification stage, classifiers of Random Forest, K-nearest neighbors, and Bagging were trained to discriminate deception and honesty.

## Results

### Action Units of Fear Can Differentiate Liars From Truth-Tellers

#### Machine Learning Classification Results

The whole dataset was split into two subsets; we arbitrarily selected 12 out of our 16 participants' data to build the model, i.e., data collected from 12 participants (42,954 frames, with 2,999 frames of lying and the rest of truth-telling) were used for training the model; and the data collected from the remaining four participants (10,832 frames in total, with 690 frames of lying and the rest of truth-telling) were used to test how accurate were the model to make new predictions. Three classifiers were trained on a dataset of 12 participants to discriminate liars from truth-tellers using feature vectors of AUs of fear (i.e., AU01, AU02, AU04, AU05, AU07, AU20, and AU26, for details of AUs of fear please, see https://imotions.com/blog/facial-action-coding-system/). All the three classifiers, Random Forest, K-nearest neighbors (IBK), and Bagging, were trained in WEKA *via* a 10-fold cross-validation procedure. In building the model, the 10-fold cross-validation procedure split all the data from the 12 participants into 10 subsets, and the algorithms were trained on 9 subsets and tested on the remaining 10th each time, repeating 10 times. When a classifier was deployed from 10-fold cross-validation, it was applied to the other four participants' data to calculate the accuracy of prediction. To highlight the relative importance of AUs of fear in classification accuracy, we eliminated all other indicators used by Beh and Goh (2019). Table 1 shows the performance of machine learning analysis, which was conducted on dataset of 12 participants and tested with the data of the remaining 4 participants.

**Table 1:** Machine learning performance of the Random Forest, IBK, and Bagging.

**Classifier**	**Accuracy (%)**	**TP Rate**	**FP Rate**	**Precision**	**Recall**	**F-Measure**	**PRC Area**	**Kappa**
Random Forest	86.9033	0.869	0.813	0.818	0.869	0.833	0.811	0.0829
IBK	85.1068	0.851	0.804	0.805	0.851	0.824	0.799	0.0624
Bagging	86.1482	0.861	0.852	0.794	0.861	0.821	0.827	0.0141

Table 1 reports the percentage of accuracy obtained on the testing dataset. In addition to accuracies, the table reports the weighted average of true-positive rate (TP rate, instances correctly classified as a given class), false-positive rate (FP rate, instances falsely classified as a given class), precision value (proportion of instances that are truly of a class divided by the total instances classified as that class), recall value (proportion of instances classified as a given class divided by the actual total in that class), F-measure (a combined measure for precision and recall), precision-recall curve (PRC) area value (a model performance metrics based on precision and recall), and kappa (which measures the agreement between predicted and observed categorizations). The details of these statistics can be seen in Witten et al. (2016).

In addition, considering the size of the dataset is relatively small, we did leave-one-person-out cross-validation (LOOCV). LOOCV utilizes each individual person as a “test” set and the remaining dataset as the training set. It is recommended for smaller datasets. The Random Forest algorithm was applied. The results showed that the average accuracy is still above 90% (*mean* = 90.16%, range from 78.74 to 95.78%).

#### The Differences of AUs of Fear Between Truth-Telling and Lying Video Clips

This analysis was carried out by examining the statistical differences of AUs of fear between truth-telling and lying video clips through paired *t*-test. To avoid the multiple-testing problem, we applied Bonferroni correction and set *p*-value to 0.007. We also calculated Cohen's d to measure effect size. The results are presented in Table 2. When bootstrapping was used, the *p*-value of comparing AU20 in the two groups was 0.006 (for AU05, the corresponding *p*-value is 0.008). This analysis revealed that liars and truth-teller had differences in the facial expressions of fear.

**Table 2:** The results of paired *t*-test for comparing the means of values of AUs of fear between truth-telling and lying video clips.

**Feature**	**Deception (Mean)**	**Truth (Mean)**	**95% CI of mean difference**	***t*-value**	***p*-value**	Effect size^*^
AU01	0.2544	0.2735	−0.1562 0.1180	−0.297	0.771	0.074
AU02	0.1308	0.1759	−0.1099 0.0196	−1.487	0.158	0.371
AU04	0.1686	0.1554	−0.0709 0.0972	0.333	0.743	0.084
AU05	0.0341	0.0639	−0.0505 −0.0090	−3.060	0.008	0.766
AU07	0.7929	0.8517	−0.3581 0.2405	−0.419	0.681	0.105
AU20	0.0838	0.1427	−0.0978 −0.0200	−3.226	**0.006**	0.807
AU26	0.3969	0.4721	−0.1825 0.0321	−1.493	0.156	0.374

* *The effect sizes were calculated by using the calculator from the website: https://memory.psych.mun.ca/models/stats/effect_size.shtml*.

### There Were More Transient Durations of AU of Fear While Lying

**Figure 3:**
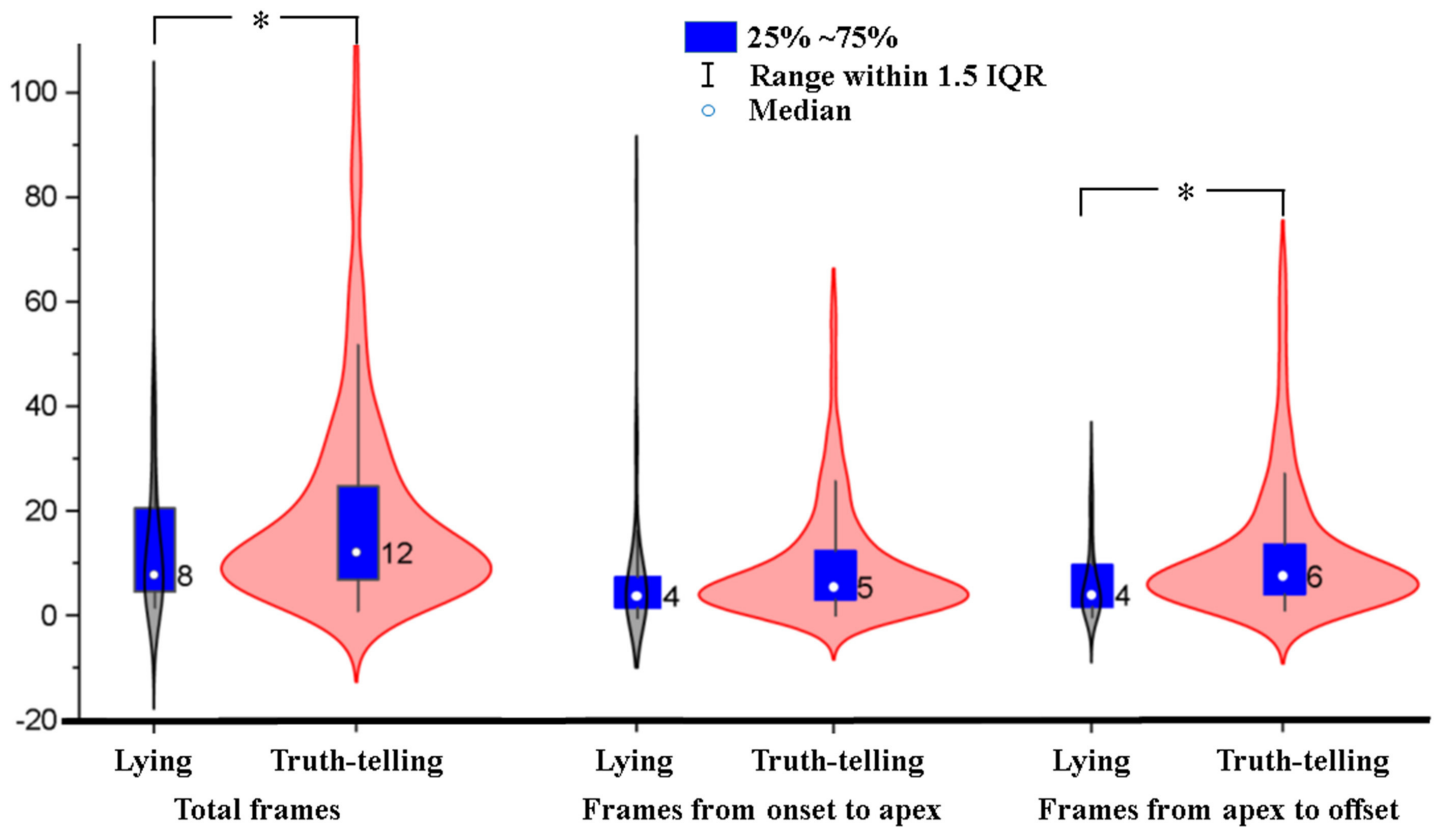
Violin plot for frames of AU20 in truth-telling and lying video clips. IQR, inter-quartile range. *statistically significant (*p* < 0.05) differences between lying and truth-telling.

Ekman (2003) reported that many people could not inhibit the activity of the AU20 (stretching the lips horizontally) while examining videotapes of people lying and telling the truth. Our results reported in section The Differences of AUs of Fear Between Truth-Telling and Lying Video Clips also found significant differences between truth-telling and lying video clips in values of AU20. Therefore, differences in the duration from onset to peak, from peak to offset, and total durations of AU20 between truth-telling video clips (in which the number of AU20 is 675) and lying video clips (in which the number of AU20 is 47) were analyzed with independent samples *t*-test, using bootstrapping with 1,000 iterations. The results showed that there were significant differences in the total duration and duration from peak to offset between truth-telling video clips and lying video clips (20.77 vs. 15.21 frames, *p* = 0.033, effect size = 0.276; 11.35 vs. 6.98 frames, *p* = 0.04, effect size = 0.347). The durations of AU20 in lying video clips were nearly four frames (133 ms) shorter than those in truth-telling video clips on average because the facial movements (herein the AU20) disappeared more quickly in the lying condition. Figure 3 shows the distribution of total frames, frames from onset to apex, and frames from apex to offset of AU20. The median is 12 in the truth-telling video clips and 8 in the lying video clips. For lying video clips, the 95% confidence interval is 10.32 to 20.11 frames for the mean of total duration, and 19.03 to 22.52 frames for truth-telling video clips. There were 16 (out of 47) AU20s whose durations were less than or equal to six frames (200 ms, one of the commonly recognized thresholds differentiating microexpressions and macroexpressions) in the lying video clips, while there were 145 (out of 675) in the truth-telling video clips. There were 32 AU20s whose durations were ≤ 15 frames (500 ms, another microexpression/macroexpression boundary, more details in discussion) in the lying video clips, and the corresponding number is 407 in the truth-telling video clips.

### Asymmetries of the Facial Movements Were More Salient in Lying Than Truth-Telling

**Figure 4:**
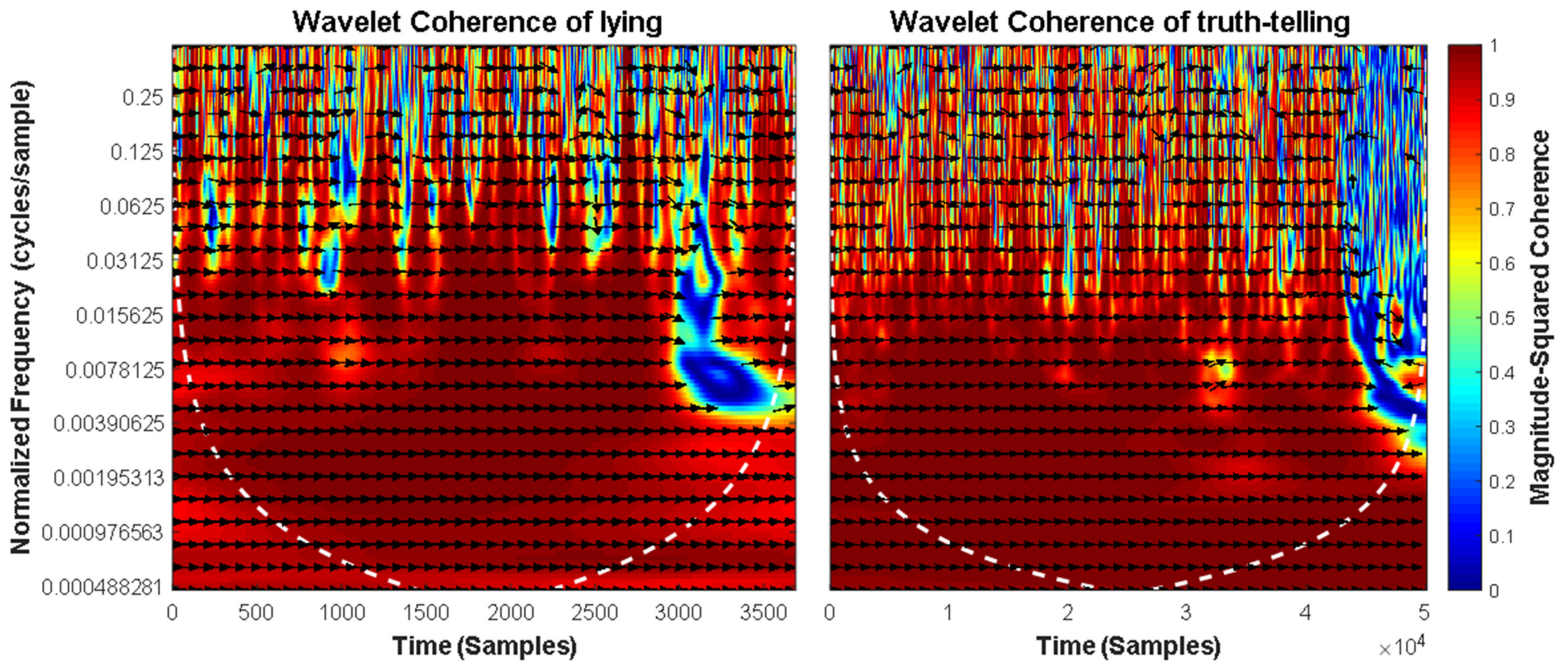
Squared wavelet coherence between the ld1 and rd1 in lying (**left** panel) and truth-telling (**right** panel) situations. The relative phase relationship is shown as arrows (a rightward arrow indicates 0 lag; a bottom-right arrow indicates a small lead of ld1; a leftward arrow indicates ld1 and ld2 are anti-correlated).

We calculated ld1 and rd1, the distance between facial landmarks predicted at the left eyebrow and left eye and the distance between those predicted at the right eyebrow and right eye (Beh and Goh, 2019) in each frame. These two distances represented movements of the left and right eyebrows. Next, we used the MATLAB function Wcohenrence (wavelet coherence) to measure the correlation between ld1 and rd1 in each video. If the movements were exactly symmetrical (e.g., they have the exact same onset time, reach the apex at the same time, and disappear at the same time), the coherence between ld1 and rd1 would be 1. Any asynchrony would result in a coherence value of <1, with a smaller coherence value indicating more asymmetry. Figure 4 shows the wavelet coherence in truth-telling and lying video clips.

The coherence outputs for each player (i.e., the average of coherence between ld1 and rd1) were then imported into the permutation test (see the following link for details: https://github.com/lrkrol/permutationTest) to compare the asymmetry differences between the lying and truth-telling situation. Permutation tests provide elegant ways to control for the overall Type I error and are distribution-free. The results showed that lying and truth-telling situations caused different coherence in facial expressions (the means of coherence are 0.7083 and 0.8096, *p* = 0.003, effect size = 1.3144).

## Discussion

The current study supported the prediction of leakage theory that leaked fear could differentiate lying from truth-telling. The results of machine learning indicated that emotional facial expressions of fear could differentiate lying from truth-telling in the high-stake game show; the paired comparisons showed significant differences between lying and truth-telling in values of AU20 of fear (AU5 is marginally significant). The results also substantiated the other two hypotheses. The duration of AUs of fear in lying was shorter than that in truth-telling, with a shorter total duration and the duration from peak to offset of AU20 of fear when lying compared to telling truth. The third hypothesis predicted that the symmetry of facial movements would be different, and the findings indicated that the facial movements were more asymmetrical in lying situations than in truth-telling situations.

In the current study, the use of machine learning classified deception and honesty. It made up the shortcomings of human coding and successfully detected the subtle differences between lying and truth-telling. Meanwhile, an objective measure of asymmetry was proposed. To our best knowledge, this is the first objective method to measure the asymmetry of facial movements. By using these methods, we were able to find differences between lying and truth-telling, which is the prerequisite for looking for clues of deception.

The machine learning approach could have some disadvantages. For example, the LOOCV is recommended for small datasets, like what we have in the current study. However, it yielded a higher variance than 10-fold cross-validation. The reason for this high variance might be that the training datasets in LOOCV have more overlap (each model was trained on an almost identical dataset), which made the outputs from different folds highly positively correlated with each other, and hence increases the overall variance (the mean of many highly correlated quantities has higher variance than does the mean of many quantities that are not as highly correlated (see James et al., 2013, p185). In our data, the variance was represented as varying accuracy rates when different participants were left out in the training set; for example, 78.74% accuracy when participant 14 was left out compared to 95.78% when participant 11 was left out. Bengio and Grandvalet (2004) argued that when independent estimates in cross-validation were correlated, the correlation that is responsible for the overall increase of variance could increase with K in a K-fold cross-validation, with leave-one-out being an extreme case where K is equal to the number of data points. In our dataset, considering the similar procedure each individual generates the same facial expression, it is highly possible that the training sets are highly correlated. Future research with a larger sample size would reduce this variance.

The leaked emotions can be cues to deception, but they are not deception *per se*. They are, however, closely linked with deception. As shown in the results, truth-tellers also experience fear. However, the dynamics of experienced fear of truth-tellers were very different from those of liars. Thus, the fear emotion could be considered as a “hot spot” of deceit. Looking for the non-verbal “hot spots” of individuals satisfies the demands of rapid evaluation. Some other approaches of deception detection, for example, brain activities, cannot provide real-time results (Vrij and Fisher, 2020). The results suggested that the “hot spots”—emotional expressions of fear—could distinguish between truthful and deceptive messages with a reasonable level of accuracy. Using machine learning, we achieved a higher accuracy (above 80%) than the average accuracy achieved by people (54%, see Bond and DePaulo, 2006). In addition, we carried out a human deception detection study (Niu, 2021), in which the video clips of the first and the last honest answering (both are from the end of the questioning to the end of the answering. We changed the durations of truth-telling video clips to keep the durations of lying and truth-telling video clips nearly same), the lying video clips are the same. Thirty college students took part in the study. The accuracy of detecting the lies was 0.34; for low-stake truth-telling video clips (the first honest answering), the accuracy of truth detection was 0.69, and for high-stake truth-telling video clips (the last honest answering), the accuracy of truth detection was 0.64, and the average of deception detection was 0.50. The results showed again that the accuracy for human deception detection was at chance level. Apart from accuracy, there was a large effect size for the AU of fear (AU20) while differentiating lies from truth.

High-stake lies were used in some previous research. For example, Vrij and Mann (2001) used the videos from media where missing people's family members announced the missing of their family and asked for help. In these videos, some of the announcers were telling the truth, while the others were hiding the truth that the people claimed to be missing were murdered by the announcers themselves. One disadvantage of these type of materials is that researchers would not have access to the truth and therefore would not be able to tell for sure if one is lying or not. Our dataset consists of high-stakes deception videos from a real game show, in which the veracity of the statements is supported by a polygraph test. That can help us achieve a relatively high ecological validity and internal validity. Considering the debate on the reliability of polygraph tests, future research could use materials where the truth is further affirmed. One example would be the game show Golden Balls, which utilizes a prisoner's dilemma setting and the truth becomes obvious after one makes a decision in the game (see Van den Assem et al., 2012).

Were the facial expressions in lying video clips all microexpressions that last for <0.2 s? The current results of total duration showed that AU20 on average lasts for 20.77 frames, i.e., 692 ms, in truth-telling video clips; and 15.21 frames, i.e., 507 ms, in lying clips. The 95% confidence intervals of total duration were from 19.03 to 22.52 frames (634–751 ms) while telling truth and were from 10.32 to 20.11 frames (344 ms ~ 670 ms) while lying. In the current study, the mean was affected by extreme values or outliers (see Figure 3). Thus, we used the median, which could be a more appropriate statistic for the duration. The median of duration in the truth-telling video clips was 12 (400 ms) and in the lying video clips was 8 (267 ms). Although the duration of (partial) fear was shorter in lying video clips than in truth-telling video clips, most of the durations in lying did not fit into the limits of traditional durations of microexpressions, i.e., <200 ms (see Shen et al., 2012). There were nearly 1/3 AU20s which durations were less than or equal to six frames (200 ms) in the lying video clips, and only 1/5 of them in the truth-telling video clips were less than or equal to six frames. By using 500 ms as the boundary between microexpressions and macroexpressions (see Matsumoto and Hwang, 2018), there were almost 2/3 of the facial expressions that could be named after microexpressions. The results suggested that the leaked emotional facial expressions in real life were much longer (the duration of the apex of leaked emotional facial expressions would be <200 ms). No matter what the duration is, or whether the facial expression is a microexpression or not, the durations of facial expressions were significantly shorter in the lying video clips than in the truth-telling video clips.

Taken together, our findings suggested that deception is detectable by using emotional facial expressions of fear in high-stake situations. Lying in high-stake situations will leak facial expressions of fear. The durations of fear were significantly different between lying and truth-telling conditions. Besides, the facial movements are more asymmetrical when one is lying than they are when one is telling the truth.

Our findings prompted that attending to the dynamic features of fear (such as symmetry and duration) can improve the ability of the people to differentiate liars from truth-teller. Besides, the machine learning approach can be employed to detect real-world deceptive behaviors, especially those high-stake ones in the situations where strong emotions are generated, associated with attempts to neutralize, mask, and fake such emotions (similar work is done in the project of iBorderCtrl, see Crampton, 2019). Certainly, the number of participants (16) in the current dataset was relatively small, which could limit the generalization of the results. We consider the current work as a preliminary exploration.

Pupil dilation and pitch of speech are found to be significantly related to deception by some studies of meta-analysis (Zuckerman et al., 1981; DePaulo et al., 2003; Levine, 2019). These cues are closely related to leakage too. The findings of Bradley et al. (2008) indicated that the pupil's changes were larger when viewing emotionally arousing pictures which also were associated with increased sympathetic activity. Pitch of speech will be different between honest and deceptive interaction (Ekman et al., 1976; Zuckerman et al., 1981). Future studies should address all these leaked clues or the “hot spots” of the deception.

## Data Availability Statement

The original contributions presented in the study are included in the article, the dataset used in the current study can be obtained on request from the first author (shenxunbing@yahoo.com).

## Ethics Statement

The studies involving human participants were reviewed and approved by the Institutional Review Board (IRB) of Jiangxi University of Traditional Chinese Medicine. The patients/participants provided their written informed consent to participate in this study. Written informed consent was obtained from the individual(s) for the publication of any potentially identifiable images or data included in this article.

## Author Contributions

XS conceived the study, conducted the experiments, analyzed the data, wrote the paper, and acquired the funding. GF analyzed the data and revised the manuscript. ZC analyzed the data. CN contributed the materials used in the current study. All authors contributed to the article and approved the submitted version.

## Conflict of Interest

The authors declare that the research was conducted in the absence of any commercial or financial relationships that could be construed as a potential conflict of interest.
